# Dissecting the transcriptional phenotype of ribosomal protein deficiency: implications for Diamond-Blackfan Anemia

**DOI:** 10.1016/j.gene.2014.04.077

**Published:** 2014-07-25

**Authors:** Anna Aspesi, Elisa Pavesi, Elisa Robotti, Rossella Crescitelli, Ilenia Boria, Federica Avondo, Hélène Moniz, Lydie Da Costa, Narla Mohandas, Paola Roncaglia, Ugo Ramenghi, Antonella Ronchi, Stefano Gustincich, Simone Merlin, Emilio Marengo, Steven R. Ellis, Antonia Follenzi, Claudio Santoro, Irma Dianzani

**Affiliations:** a Department of Health Sciences, University of Eastern Piedmont, Novara, Italy; b Department of Sciences and Technological Innovation, University of Eastern Piedmont, Alessandria, Italy; c Department of Chemistry, University of Milan, Italy; d U1009, AP-HP, Service d'Hématologie Biologique, Hôpital Robert Debré, Université Paris VII-Denis Diderot, Sorbonne Paris Cité, F-75475 Paris, France; e New York Blood Center, NY, USA; f International School for Advanced Studies (SISSA/ISAS), Trieste, Italy; g Department of Pediatric Sciences, University of Torino, Torino, Italy; h Department of Biotechnologies and Biosciences, Milano-Bicocca University, Italy; i University of Louisville, KY, USA

**Keywords:** DBA, Diamond Blackfan anemia, RP, ribosomal protein, RS, ribosomal stress, PCA, principal component analysis, PC, principal component, GO, gene ontology, Ribosomal protein, Diamond Blackfan Anemia, Ribosomopathy, Bone marrow failure

## Abstract

Defects in genes encoding ribosomal proteins cause Diamond Blackfan Anemia (DBA), a red cell aplasia often associated with physical abnormalities. Other bone marrow failure syndromes have been attributed to defects in ribosomal components but the link between erythropoiesis and the ribosome remains to be fully defined. Several lines of evidence suggest that defects in ribosome synthesis lead to “ribosomal stress” with p53 activation and either cell cycle arrest or induction of apoptosis. Pathways independent of p53 have also been proposed to play a role in DBA pathogenesis.

We took an unbiased approach to identify p53-independent pathways activated by defects in ribosome synthesis by analyzing global gene expression in various cellular models of DBA. Ranking-Principal Component Analysis (Ranking-PCA) was applied to the identified datasets to determine whether there are common sets of genes whose expression is altered in these different cellular models. We observed consistent changes in the expression of genes involved in cellular amino acid metabolic process, negative regulation of cell proliferation and cell redox homeostasis.

These data indicate that cells respond to defects in ribosome synthesis by changing the level of expression of a limited subset of genes involved in critical cellular processes. Moreover, our data support a role for p53-independent pathways in the pathophysiology of DBA.

## Highlights

• Ribosomopathies such as DBA are caused by ribosome dysfunction that activates p53.
• p53-independent pathways may suggest possible treatments for DBA.
• Expression analysis was performed in three p53-null models of DBA.
• Genes involved in apoptosis and cell redox homeostasis were especially affected.
• DBA is due to cumulative effects of p53-dependent and independent pathways.

## 1 Introduction

Mutations in genes encoding ribosomal proteins result in Diamond Blackfan Anemia (DBA), a bone marrow failure syndrome characterized by pure erythroid aplasia (Draptchinskaia et al., 1999; Vlachos et al., 2008). In addition to bone marrow failure, malformations are observed in approximately one third of the patients. DBA is inherited with an autosomal dominant pattern and results from haploinsufficiency for single ribosomal proteins (RPs). To date eleven genes encoding ribosomal proteins have been found mutated in DBA patients, i.e. *RPS19*, *RPS24*, *RPS17*, *RPL5*, *RPL11*, *RPS7*, *RPL35A*, *RPS26*, *RPS10*, *RPL26*, and *RPL15* (Boria et al., 2010; Draptchinskaia et al., 1999; Gazda et al., 2012; Landowski et al., 2013; Quarello et al., 2010).

In addition to DBA several other ribosomopathies have been described (Narla and Ebert, 2010). Many of these are bone marrow failure syndromes but other ribosomopathies where hematopoiesis is unaffected have also been identified (Freed et al., 2010). The DBA phenotype has been ascribed to a peculiar sensitivity of the erythron and tissues of the developing embryo to haploinsufficiency for ribosomal proteins. This hypothesis is based on information obtained using both cellular models and model organisms. Deficiencies in factors involved in ribosome synthesis have been studied extensively in Drosophila, Xenopus, zebrafish and mouse (Danilova et al., 2008; Kongsuwan et al., 1985; McGowan et al., 2008; Miller and Gurdon, 1970). These defects cause the induction of a cellular stress response, called ribosomal (or nucleolar) stress (RS) that results in activation of p53-dependent and independent pathways, which block proliferation and/or induce apoptosis (Dutt et al., 2011; Moniz et al., 2012; Torihara et al., 2011). Whereas pharmacological or genetic inhibition of p53 is able to attenuate phenotypes in many of these models, treatment based on p53 inhibition appears unrealistic in humans because of attendant cancer risks.

To shed light into pathways that are activated by ribosomal stress in human cells expressing reduced levels of ribosomal proteins we have studied the transcriptome of three different cellular models of DBA looking for intersecting patterns of gene expression changes.

## 2 Design and methods

### 2.1 Cell cultures

Human erythroleukemia cell line TF1 (ATCC Number: CRL-2003) was grown in RPMI 1640 medium supplemented with 10% FBS, 2 mM l-glutamine, 100 UI/mL penicillin, 100 μg/mL streptomycin and 5 ng/mL GM-CSF. TF1 cells expressing inducible shRNAs against RPS19 or a scrambled shRNA were provided by Dr. Stefan Karlsson (Miyake et al., 2005) (shRNAs SCR, B and C). shRNA expression was induced by 0.5 μg/mL doxycycline (DOX) for four days. TF1 cells for transduction were thawed and maintained for minimum two passages before being transduced with lentivirus prrl-shSCR or prrl-shRPL5A or prrl-shRPL11A (Moniz et al., 2012) with an MOI of 10. Two days after transduction, Green Fluorescent Protein (GFP) positive cells were sorted by flow cytometry and cultured under the same conditions for four days.

For qRT-PCR validation and flow cytometric analysis we also designed and produced a third generation lentiviral vector (LV) system expressing scrambled or RPS19 shRNA both of them co-expressing GFP under the control of the human PGK promoter (Miyake et al., 2005) (shRNAs SCR and C). LVs were obtained after transient transfection of 293T cells by the calcium phosphate method (Taulli et al., 2005) with the packaging plasmids (pMDLg/pRRE, pRSV-REV and pMD2-VSVG) and the transfer vectors expressing either the scrambled or the RPS19 shRNA. TF1 cells were transduced with MOI 10 the described LVs (Follenzi et al., 2000). Transduction efficiency was evaluated after three days by GFP detection. Cells were collected for analysis four days after transduction.

### 2.2 TP53 analysis

Genomic DNA was isolated from TF1 cells using a QIAamp DNA Mini kit (Qiagen) according to the manufacturer's protocol. Primers were designed to amplify exons 4–9 and their flanking regions. PCR was performed using AmpliTaq Gold DNA Polymerase (Applied Biosystems) and amplicons were sequenced in both directions using a Big Dye Terminator® v1.1 cycle sequencing kit (Applied Biosystem) and an ABI PRISM® 3100 genetic analyzer. Total RNA was isolated from TF1 cells using a RNeasy Plus Mini kit (Qiagen) and reverse transcribed with a High Capacity cDNA Reverse Transcription kit (Applied Biosystems). *TP53* was amplified from cDNA and sequenced. Sequencing of *TP53* from primary CD34^+^ cells was performed in parallel as a wild type control.

For the nuclear localization assay TF1 cells were lysed as previously described (Andrews and Faller, 1991) and subjected to western blot analysis.

### 2.3 Western blot

Cells were lysed in Lysis Buffer (50 mM Tris–HCl pH 8, 1 mM EDTA, 150 mM NaCl, 0.5% NP-40) supplemented with protease inhibitors. Cell debris was removed by centrifugation at 13,000 *g* for 10 min and the supernatant was collected. Proteins were separated on 12% SDS–PAGE, transferred on nitrocellulose membrane and incubated with antibodies specific for RPS19 (Abnova), RPL5 (Abcam), RPL11 (Invitrogen), β-actin (Sigma), p53, nucleolin and GAPDH (Santa Cruz Biotechnology). Detection of immunoblots was carried out with Western Lightning® Plus-ECL (PerkinElmer). Downregulation or overexpression of the proteins of interest was estimated after normalization to the intensity of GAPDH or β-actin.

### 2.4 Flow cytometry

Analysis of maturation markers was performed on TF1 cells four days after transduction with SCR or RPS19 shRNAs. 5 × 10^4^ cells were incubated for 15 min with PE-conjugated antibodies specific for CD117 (c-KIT), CD34, CD71 and CD235a (glycophorin A). Cells were then washed with PBS and examined using a flow cytometer (FACSCalibur, Becton-Dickinson). Cell cycle analysis was performed using propidium iodide (PI) staining. Briefly, cells were fixed, treated with RNase A and stained with PI 40 μg/mL, then subjected to flow cytometry analysis.

### 2.5 RNA isolation and microarray processing

Total RNA for microarray analysis was isolated using either a TRIzol® reagent (Invitrogen) or a RNeasy Plus Mini kit (Qiagen) according to the protocols supplied by the manufacturers. RNA quantification, quality assessment and labeling were performed as described in Avondo et al. (2009). Labeled cRNA was hybridized on Affymetrix GeneChip Human Genome U133A 2.0 Arrays. Microarray processing and data analysis were performed as described by Avondo et al. (2009).

### 2.6 Ranking-Principal Component Analysis (Ranking-PCA)

PCA (Massart et al., 1988, 1998) is a multivariate pattern recognition method that allows the representation of the original dataset in a new reference system characterized by new variables called principal components (PCs). By the use of a restricted number of significant PCs, experimental noise and random variations can be eliminated. PCA is exploited in Ranking-PCA (Marengo et al., 2010; Polati et al., 2012; Robotti et al., 2011) to select the most discriminating variables (i.e. candidate biomarkers) between two groups of samples (e.g. control vs. pathological) and sort them according to their decreasing discrimination ability. Here, Ranking-PCA was applied by calculating PCs in leave-one-out (LOO) cross-validation. The analysis we performed aimed to identify the transcriptome abnormalities found in human TF1 cells with a defect of RPS19, RPL5 or RPL11.

**Fig. 1:**
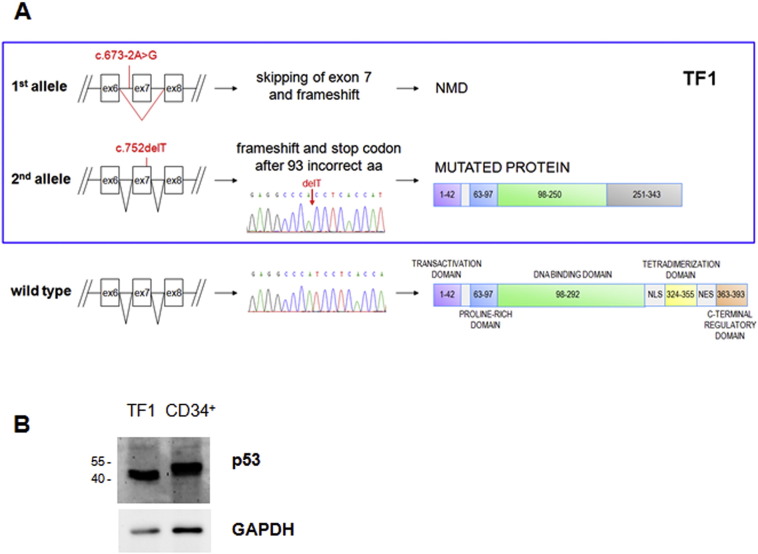
p53 in TF1 cells. A. TF1 cells do not present the wild type form of p53. Sequencing of genomic DNA showed two mutations in trans: one leads to the skipping of exon 7 and nonsense mediated mRNA decay (NMD), the other induces frameshift without NMD and was also detected by cDNA sequencing, as shown in the electropherogram. The aberrant transcript gives rise to a mutant protein that carries 93 incorrect amino acids at the C-terminus. Electropherogram of p53 from CD34^+^ primary cells and a schematic representation of p53 protein domains are shown as a wild type control. B. Immunoblotting performed with an antibody against the N-terminal region of p53 reveals a smaller protein in TF1 cells than the full-length p53 expressed by CD34^+^ cells.

The dataset consisted of measurements from two sets of experiments:

- TF1 cell lines with downregulation of RPS19 (labeled *S19* in Fig. 1) and their SCR controls (labeled *CS*);
- TF1 cell lines downregulated for RPL5 and RPL11 (labeled *L5* and *L11* respectively) and their scrambled controls (labeled *CL*).

Since the datasets were not directly comparable, they were independently mean centered (i.e. the average value of each variable is subtracted from each sample for each dataset separately). Then, Ranking-PCA was applied to the TF1 dataset consisting in 17 samples (7 control and 10 pathological) described by 10,194 variables (probes). Only the first PC was selected and provided the correct classification of all the samples, as assessed by calculation of the percent non-error-rate (NER%), defined as the percentage of correct assignments (NER% = 100%).

The performance of Ranking-PCA was compared to other classification tools as Partial Least Squares-Discriminant Analysis (PLS-DA) (Marengo et al., 2008) obtaining similar classification performances but Ranking-PCA provides an exhaustive set of candidate biomarkers ranked according to their decreasing discriminant ability.

### 2.7 Quantitative RT-PCR

For qRT-PCR analysis total RNA was isolated using TRIzol® reagent. cDNA was synthesized using the High Capacity cDNA Reverse Transcription Kit (Applied Biosystems). Quantitative PCR was performed with an Abi Prism 7000 instrument (Applied Biosystems) using Taqman® Gene Expression Assays (Applied Biosystems). PCR reactions were run in triplicate. Ct values were normalized to GAPDH or β-actin, used as endogenous controls, and expression levels were calculated with the ddCt method (Livak and Schmittgen, 2001). Fold changes in the expression of the target gene were equivalent to 2^− ddCt^. Fold change data are presented as mean ± SD. Data were analyzed with Student's t-test.

## 3 Results

### 3.1 Characterization of TF1 cell lines

RPS19-silenced TF1 cells have been widely employed to investigate DBA pathophysiology (Badhai et al., 2009; Flygare et al., 2007; Miyake et al., 2005, 2008). Using this cell model it was demonstrated for the first time that human RPS19 is required for the maturation of 40S ribosomal subunits (Flygare et al., 2007). When RPS19-deficient TF1 cells were treated with erythropoietin (EPO), significant suppression of erythroid differentiation, cell growth, and colony formation was observed (Miyake et al., 2005), along with the increase of apoptotic cells (Miyake et al., 2008). These previous studies did not ascertain the status of p53, whereas more recent investigations have pointed out that ribosomal stress activates both p53 dependent and independent pathways. To address this issue we sequenced the *TP53* gene in both parental and RPS19 downregulated TF1 cell lines (Miyake et al., 2005). Sequencing of genomic DNA showed two mutations in trans. On one allele, mutation c.673-2A>G in the acceptor splice site of exon 7 is expected to lead to the skipping of this exon and to nonsense mediated mRNA decay (NMD, Fig. 1A), as confirmed by the absence of this transcript in cDNA sequencing analysis (data not shown).

On the other allele, we detected mutation c.752delT, already described by Urashima et al. (1998). We found that this mutation induces frameshift without NMD, since the stop codon of the new reading frame is located in proximity of the last splicing site. This mutation was also detected in p53 mRNA expressed by TF1 cells, as shown by cDNA sequencing (Fig. 1A). The aberrant transcript gives rise to a protein with 93 incorrect amino acids at the C-terminus and with a predicted size of approximately 38 kDa (Fig. 1A). Accordingly, immunoblotting performed with an antibody against the N-terminal region of p53 revealed a smaller protein in TF1 cells than the full-length p53 expressed by CD34^+^ cells (Fig. 1B). This protein lacks the nuclear localization signal and part of the DNA binding domain, therefore it accumulates in the cytoplasm (Fig. S1) and is presumably inactive. The presence of null mutations on both alleles of p53 makes TF1 cells a suitable model for the investigation of p53-independent pathways activated by ribosomal stress.

### 3.2 Phenotypic characterization of RPS19 downregulated cells

**Fig. 2:**
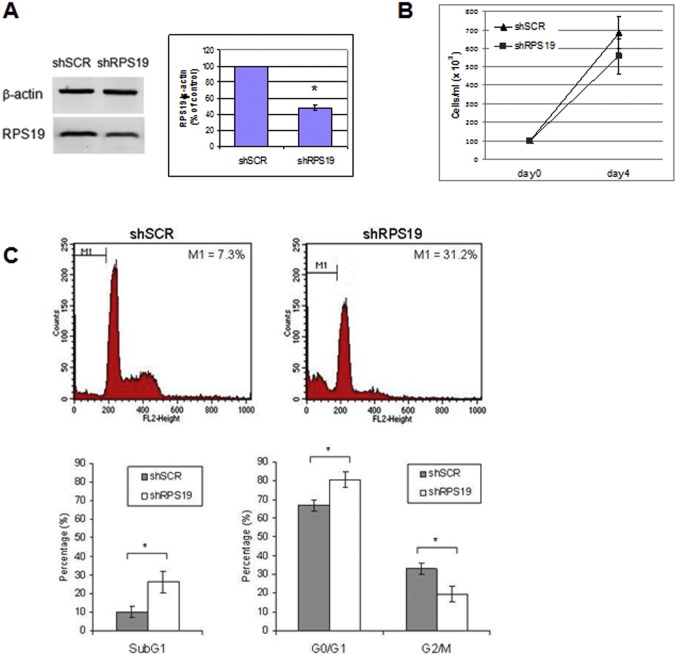
RPS19 silencing in TF1 cells. A. Western blot showing the downregulation of RPS19 protein in TF1 cells, compared to scrambled controls, after four days of DOX treatment. The densitometry analysis, performed on three replicates, shows a statistically significant downregulation of RPS19. *p value < 0.05. B. Growth curve of TF1 cells treated with DOX for four days. C. Cell cycle analysis by flow cytometry of TF1 cells treated with DOX for four days and stained with propidium iodide. The bar graphs show the percentage of cells in subG1 phase on total cells and the percentage of cells in G_0_/G_1_ and G2/M phase on viable cells, as the mean of three replicates. Standard deviation bars are shown. *p value < 0.05.

We then investigated how RPS19 downregulation affected proliferation, apoptosis and maturation in TF1 cells cultured without EPO. Cells expressing shRNA against RPS19 were examined after four days of DOX treatment and compared to a scrambled (SCR) control. The level of RPS19 protein was reduced to about 50% (Fig. 2A), thus mimicking RP haploinsufficiency showed by DBA patients, who always carry the deleterious mutation in heterozygosity. We observed a slight, not significant decrease in proliferation (Fig. 2B).

Propidium iodide staining revealed a significant increase in the subG1 population which includes late-stage apoptotic and necrotic cells. Among viable cells, a large proportion of RPS19 silenced cells were in G_0_/G_1_ phase, whereas the percentage of cells in G2/M phase decreased about 1.7 fold compared to SCR control (Fig. 2C).

We then characterized the phenotypic expression of surface markers by flow cytometry. TF1 cells were transduced with a lentivirus expressing SCR or RPS19 shRNAs and GFP as a reporter gene. The transduction efficiency was higher than 97% (Fig. S2A). In this model, constitutively expressing shRNAs, RPS19 downregulation, as well as its effects on proliferation and cell cycle, was very similar to the DOX-inducible model (data not shown). The proportion of cells positive for two early hematopoietic markers, c-KIT and CD34, and for two markers specific for erythroid differentiation, CD71 and glycophorin A, was unchanged (Fig. S2B).

### 3.3 Gene expression profiling of cells with RP deficiency

To identify p53-independent pathways activated by a RP defect, we used three TF1 cell lines expressing shRNAs against RPS19, RPL5 or RPL11, the three most frequently mutated DBA genes. The downregulation of the respective ribosomal proteins was assessed by western blotting (Figs. 2A, S3). The observed downregulation of RPL5 was about 40% and that of RPL11 was about 70%, as compared with scrambled controls.

We analyzed whole genome expression profiles of the three TF1 cell lines downregulated for RPS19, RPL5 or RPL11 (named hereafter TF1 shRPS19, TF1 shRPL5, TF1 shRPL11) as compared to SCR controls. The expression study was performed using Affymetrix GeneChip Human Genome U133A 2.0 Arrays which allow the screening of 18,400 transcripts. Each dataset showed a decrease in the transcript corresponding to the downregulated RP (fold change RPS19: 0.12; RPL5: 0.26; RPL11: 0.11).

**Fig. 3:**
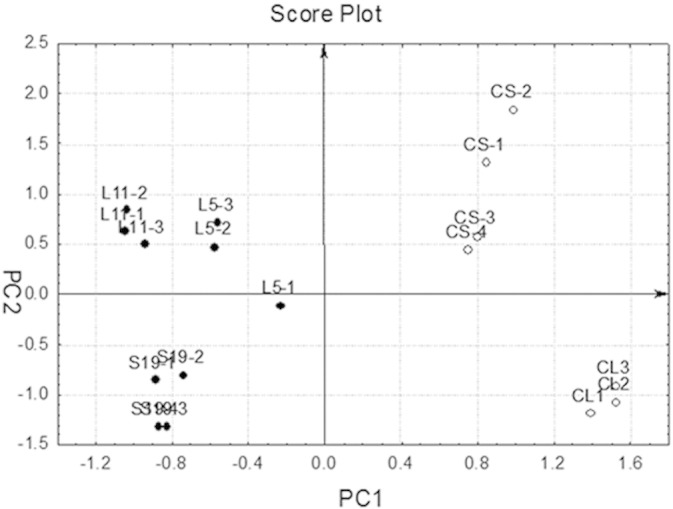
PCA on RP deficient TF1 cells. Score plot of the first two PCs calculated on the dataset containing TF1 cell lines downregulated for RPS19, RPL5 and RPL11. Samples are separated along PC_1_ in controls (positive scores; empty circles) and pathological (negative scores; full circles). Labels: *S19* = TF1 downregulated for RPS19; *CS* = scrambled controls for RPS19; *L5* and *L11* = TF1 downregulated for RPL5 and RPL11; *CL* = scrambled controls for RPL5 and RPL11.

In order to identify the transcriptional signature of RP deficiency in p53-deficient cells we intersected the three TF1 cell lines downregulated for RPS19, RPL5 and RPL11 using Ranking-PCA. Ranking-PCA is a statistical method that can select and sort the most discriminating variables between groups of pathological and control samples (Robotti et al., 2011). Fig. 3 represents the results of PCA performed on the first 205 variables selected by Ranking-PCA. The first PC accounts for about 79% of the overall information. The selected variables are reported in Table S1 according to the order in which they were included in the Ranking-PCA model. It is important to note that the results obtained by Ranking-PCA do not necessarily include all the genes that have the highest fold change in RP-deficient cells as compared to their controls. Instead the analysis is carried out to provide the set of dysregulated genes common to the three TF1 cell lines silenced for RPS19, RPL5 or RPL11: a gene is added only if it shows a similar dysregulation in all datasets. Although PC_2_ is responsible for only about 4% of the total information, it does reflect effects of the pathology since control and pathological samples from the same cell line (TF1-S and TF1-L cell lines) lie at opposite values along this PC. PC_1_ and PC_2_ together are able to clearly distinguish the four groups of samples corresponding to two different downregulation models (TF1-S is an inducible model, whereas L is a constitutive downregulation model), both control and pathological, and to RPs pertaining to different ribosome subunits.

### 3.4 Biological processes altered in cells with RP deficiency

In order to systematically detect impaired biological processes of these cells, we analyzed the genes included in the Ranking-PCA list by employing the tool of gene annotation provided by DAVID (Database for Annotation, Visualization and Integrated Discovery) at http://david.abcc.ncifcrf.gov/. The results included classifications according to Gene Ontology (GO) and PANTHER databases. GO categories for Biological Processes showed an enrichment, among others, of genes involved in cellular amino acid metabolic process, negative regulation of cell proliferation, apoptosis and cell redox homeostasis (Table 1). The PANTHER Biological Process annotation identified statistically significant over-representation of genes involved in hematopoiesis and in amino acid and steroid metabolism (Table 2).

Two genes stood out whose expression was increased in this analysis, EPOR and TFRC, whereas another noteworthy gene, SOD2, displayed reduced expression (Table S1).

Among the differentially expressed genes, there were only four genes whose transcription could be activated by p53 (Riley et al., 2008): APAF1, FDXR, SCD and PYCARD. The first three genes were downregulated, whereas the proapoptotic gene PYCARD showed a higher level in RPS19 silenced TF1 cells. As expected, the vast majority of known p53 targets (Riley et al., 2008) did not show an altered expression in RP depleted TF1 cells. Our data suggest that the increased expression of PYCARD may be mediated by p53 independent pathways.

### 3.5 Quantitative RT-PCR validation of microarray data

**Fig. 4:**
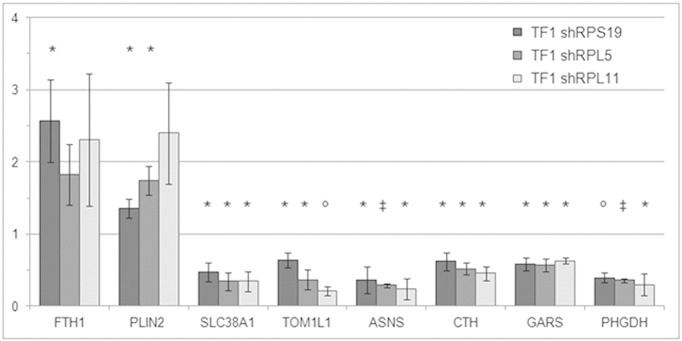
Validation of microarray results by qRT-PCR. Fold change of the expression of eight altered genes in RP depleted TF1 cells compared to scrambled controls (set equal to 1). Data were obtained by qRT-PCR measurement and normalized to GAPDH or β-actin levels. *p value < 0.05, ^○^p < 0.01, ^‡^ p < 0.001.

In order to corroborate the microarray gene expression results, we selected eight genes among the top genes of the Ranking-PCA list or among those highlighted by the PANTHER analysis. Real-time RT-PCR was performed on the same RNA samples used for microarray analysis (TF1 shRPL5, TF1 shRPL11) or on different samples with a similar level of RP downregulation (TF1 shRPS19, both DOX-inducible model and transduced cells constitutively expressing shRNAs). The expression level of FTH1 and PLIN2 (up-regulated in RP defective cells) and SLC38A1, TOM1L1, ASNS, CTH, GARS and PHGDH (down-regulated in RP defective cells) was tested. All genes were found concordantly dysregulated in RP depleted cells compared to scrambled controls (Fig. 4). These data imply that the expression patterns detected by microarray analysis are in good agreement with those detected by qRT-PCR and validate our conclusions.

## 4 Discussion

Many lines of evidence have underscored the pivotal role of p53 activation in the induction of cell death and proliferation block in cells and organisms subjected to ribosomal stress (Danilova et al., 2008; Dutt et al., 2011; Ellis and Gleizes, 2011; McGowan et al., 2008). The decrease in p53 activity by genetic means or using chemical inhibitors has proven useful to attenuate the proapoptotic phenotype of these models. However, p53 inhibitors cannot be used in the therapy of patients with DBA because they would drastically increase their cancer risk. The identification of p53-independent pathways that are induced by ribosomal stress may suggest new druggable steps that could be modulated to reduce the phenotypic consequences of ribosomal protein haploinsufficiency.

The aim of our work was to identify the p53-independent cellular processes that are altered during ribosomal stress due to deficiency of DBA RPs. To this aim we have used human TF1 cell lines that were silenced for the three RPs that are most commonly mutated in DBA patients, i.e. RPS19, RPL5 or RPL11. In fact, TF1 cells carry deleterious mutations on both p53 alleles, which abolish p53 function, as shown by sequencing and functional studies.

To search for impaired processes we have intercepted the transcriptomes of the three TF1 cell lines using Ranking-PCA. We identified genes involved in cell proliferation and apoptosis, in agreement with a previous study that showed abnormal levels of apoptosis related proteins in TF1 cells downregulated for RPS19 (Miyake et al., 2008). We detected the upregulation of PYCARD, a transcript encoding a proapoptotic protein that triggers the activation of caspases (Ohtsuka et al., 2004). Overexpression of Pycard in mouse inhibits the proliferation of erythroid cells, promotes their apoptosis, and interferes with their terminal differentiation (Hu et al., 2011). Abnormal expression of genes related to apoptosis was also reported in bone marrow CD34^+^ cells isolated from three DBA patients with mutations in RPS19 and in remission from the disease (Gazda et al., 2006), and in a previous study by our group focused on unraveling the gene expression alterations in fibroblasts isolated from DBA patients with RPS19 mutations (Avondo et al., 2009).

Moreover, a large cluster of significantly underexpressed RPs was described in these two reports (Avondo et al., 2009; Gazda et al., 2006). On the contrary, both the present study and a previous one which examined RPS19-deficient TF1 cells showed normal levels of RP mRNAs, with the exception of RPL3 (Badhai et al., 2009, Table S1). This lack of congruence might be explained by the presence or absence of wt p53 in primary cells and TF1 model, respectively. In fact, it is known that p53 can inhibit mTORC1 (Hasty et al., 2013), which mediates the transcription of RP genes (Xiao and Grove, 2009).

The expression of several genes involved in erythroid maturation is increased, in particular, erythropoietin receptor (EPOR), transferrin receptor (TFRC), CDKN2A, that encodes for p16, whose transcriptional upregulation in progenitor cells promotes differentiation (Minami et al., 2003), and HOXB2, a target of the erythroid transcription factor GATA1 (Vieille-Grosjean and Huber, 1995). However, maturation is not altered in these cells in our experimental conditions, as shown by the immunophenotypic analysis of RPS19 downregulated TF1 cells.

Interestingly, enrichment of genes involved in hematopoiesis and cell redox homeostasis was observed. Our study shows a downregulation of certain genes that participate in the protection against oxidative stress, in particular superoxide dismutase 2 (SOD2) and thioredoxin reductase 1 (TXNRD1) in cells depleted of RPs. A reduced expression of SOD2 was observed also in RPL11-deficient zebrafish (Danilova et al., 2011). These results indicate that cells depleted of RPs may have an enhanced sensitivity to oxidative stress. The same phenomenon has been suggested for two other bone marrow failure syndromes, i.e. Fanconi Anemia (FA) and Shwachman-Diamond Syndrome (SDS). This sensitivity may lead to increased apoptosis and decreased cell growth (Ambekar et al., 2010; Bogliolo et al., 2002; Mukhopadhyay et al., 2006).

Finally, we found dysregulation of clusters of genes involved in amino acid metabolism and lipid metabolism. Downregulation of genes involved in biosynthetic processes has been reported also in zebrafish with a RPL11 deficiency (Danilova et al., 2011).

All these data show that when a RP is defective there is a set of biological functions/molecular processes that are affected in different types of human cells, either primary cells from DBA patients or experimental models. The increased destruction of erythroid progenitors observed in patients with DBA may be due to the cumulative effects of p53-dependent and -independent pathways. Cells that undergo ribosomal stress alter the expression profile of a set of genes, which are consistent with the pro-apoptotic and hypo-proliferative phenotype. Further studies are needed to ascertain whether antioxidant treatment may relieve the DBA phenotype in vitro.

## Conflict of interest statement

The authors declare no conflicts of interest.

## Figures and Tables

**Table 1:** Genes included in the PC1 were annotated using Gene Ontology biological process.

Term	Count	p value	Genes
GO:0008610 — lipid biosynthetic process	19	4.12E − 08	FCER1A, EBP, SPTLC2, SCD, HMGCS1, FDXR, LTC4S, SC4MOL, FDFT1, FAR2, PIGK, PIGF, LPCAT1, SH3GLB1, DHCR7, PBX1, LTA4H, SC5DL, NSDHL
GO:0016053 — organic acid biosynthetic process	12	1.99E − 06	FCER1A, C8ORF62, SCD, ASNS, LTC4S, SC4MOL, CTH, GOT1, SH3GLB1, PHGDH, LTA4H, PSAT1, SC5DL
GO:0016126 — sterol biosynthetic process	7	2.84E − 06	EBP, DHCR7, HMGCS1, SC5DL, FDFT1, SC4MOL, NSDHL
GO:0006694 — steroid biosynthetic process	9	6.53E − 06	EBP, DHCR7, HMGCS1, FDXR, PBX1, SC5DL, FDFT1, SC4MOL, NSDHL
GO:0043436 — oxoacid metabolic process	21	7.41E − 06	FCER1A, C8ORF62, SCD, CS, GARS, EPRS, ASNS, LTC4S, PCK2, SLC7A5, SC4MOL, MTHFD2, CTH, GOT1, SH3GLB1, GFPT1, PHGDH, LTA4H, DDAH2, PSAT1, SC5DL, ALDH9A1
GO:0044106 — cellular amine metabolic process	14	5.98E − 05	C8ORF62, GARS, EPRS, ASNS, SLC7A5, CTH, GOT1, GFPT1, PHGDH, SMOX, PAFAH1B1, AMD1, PSAT1, DDAH2, ALDH9A1
GO:0044255 — cellular lipid metabolic process	16	0.0013	FCER1A, SPTLC2, SCD, HMGCS1, PIP5K1B, LTC4S, SC4MOL, FDFT1, PIGK, PIGF, LPCAT1, SH3GLB1, LTA4H, PAFAH1B1, SC5DL, NR1H3
GO:0006520 — cellular amino acid metabolic process	10	0.0014	C8ORF62, CTH, GOT1, GFPT1, GARS, PHGDH, EPRS, ASNS, PSAT1, DDAH2, SLC7A5
GO:0006633 — fatty acid biosynthetic process	6	0.0023	FCER1A, SCD, LTA4H, LTC4S, SC5DL, SC4MOL
GO:0009309 — amine biosynthetic process	6	0.0026	C8ORF62, CTH, GOT1, PHGDH, ASNS, PSAT1, AMD1
GO:0008202 — steroid metabolic process	9	0.0026	EBP, DHCR7, HMGCS1, FDXR, PBX1, SC5DL, FDFT1, SC4MOL, NSDHL
GO:0006575 — cellular amino acid derivative metabolic process	8	0.0034	CTH, PHGDH, PAFAH1B1, SMOX, AMD1, ALDH9A1, SOD2, GLRX2
GO:0008203 — cholesterol metabolic process	6	0.0044	EBP, DHCR7, HMGCS1, FDXR, FDFT1, NSDHL
GO:0010243 — response to organic nitrogen	5	0.0063	ALDOC, HMGCS1, ASNS, PPP3CA, DDIT3
GO:0019725 — cellular homeostasis	13	0.0086	CLNS1A, FTH1, DDIT3, SOD2, GLRX2, LOC100130902, TFRC, FTHL3, FTHL16, EPOR, TXNRD1, PPP3CA, SH3BGRL3, SLC39A4, EIF2B4, FTHL20, ADD1
GO:0008285 — negative regulation of cell proliferation	11	0.0099	CEBPA, LST1, FTH1, SOD2, MAGED1, CTH, CDKN2A, FTHL3, BTG3, MYO16, FTHL16, ASPH, EMP3, FTHL20
GO:0006915 — apoptosis	15	0.0109	DPF2, ALDOC, LGALS1, SOD2, TRADD, GLRX2, MAGED1, CDKN2A, SHARPIN, SH3GLB1, BRE, PYCARD, AVEN, APAF1, TRAF3
GO:0006259 — DNA metabolic process	13	0.0156	GLRX2, MCM6, SOD2, TFAM, CDKN2A, CSNK1D, RRM1, MUS81, BRE, APAF1, OGG1, TRIP13, RBMS1
GO:0043450 — alkene biosynthetic process	3	0.0204	FCER1A, LTA4H, LTC4S
GO:0006644 — phospholipid metabolic process	7	0.0241	PIGK, PIGF, LPCAT1, SH3GLB1, PIP5K1B, PAFAH1B1, FDFT1
GO:0006691 — leukotriene metabolic process	3	0.0269	FCER1A, LTA4H, LTC4S
GO:0006732 — coenzyme metabolic process	6	0.0338	MTHFD2, CTH, PANK3, CS, SOD2, GLRX2
GO:0021570 — rhombomere 4 development	2	0.0347	HOXA1, HOXB2
GO:0006461 — protein complex assembly	12	0.0348	TFAM, CTH, TSPAN4, ALDOC, IRF7, RRM1, EPRS, TUBA4A, HSPA4, WIPF1, SURF1, SOD2
GO:0030262 — apoptotic nuclear changes	3	0.0367	CDKN2A, SHARPIN, APAF1
GO:0044271 — nitrogen compound biosynthetic process	9	0.0371	CEBPA, C8ORF62, CTH, GOT1, RRM1, PHGDH, ASNS, PSAT1, DDAH2, AMD1
GO:0045454 — cell redox homeostasis	4	0.0374	LOC100130902, TXNRD1, SH3BGRL3, DDIT3, GLRX2
GO:0046486 — glycerolipid metabolic process	6	0.0416	PIGK, PIGF, SH3GLB1, PIP5K1B, PAFAH1B1, NR1H3
GO:0006749 — glutathione metabolic process	3	0.0421	CTH, SOD2, GLRX2
GO:0021610 — facial nerve morphogenesis	2	0.0460	HOXA1, HOXB2
GO:0021569 — rhombomere 3 development	2	0.0460	HOXA1, HOXB2
GO:0021604 — cranial nerve structural organization	2	0.0460	HOXA1, HOXB2
GO:0021612 — facial nerve structural organization	2	0.0460	HOXA1, HOXB2
GO:0009888 — tissue development	14	0.0479	S100A4, TRIM15, LOC100130902, CDKN2A, HOXB2, SHARPIN, GFPT1, SEMA3C, EPOR, TXNRD1, PBX1, APAF1, CA2, PPP3CA, NSDHL
GO:0006650 — glycerophospholipid metabolic process	5	0.0496	PIGK, PIGF, SH3GLB1, PIP5K1B, PAFAH1B1

**Table 2:** Genes included in the PC1 were annotated using Panther.

Term	Count	p value	Genes
BP00297: other steroid metabolism	3	0.0048	SC5DL, FDFT1, SC4MOL
BP00026: cholesterol metabolism	5	0.0054	EBP, HMGCS1, FDFT1, SC4MOL, NSDHL
BP00284: hematopoiesis	5	0.0063	CEBPA, STAP1, EPOR, PBX1, TRIM15
BP00013: amino acid metabolism	8	0.0085	C8ORF62, CTH, GOT1, SLC7A1, CS, PHGDH, ASNS, PSAT1, SLC7A5
BP00014: amino acid biosynthesis	4	0.0122	C8ORF62, CS, PHGDH, ASNS, PSAT1
BP00295: steroid metabolism	6	0.0314	EBP, HMGCS1, SC5DL, FDFT1, SC4MOL, NSDHL
