# Supplementary material for: Dissecting the transcriptional phenotype of ribosomal protein deficiency: implications for Diamond-Blackfan Anemia
Source: Gene. 2014 Jul 25;545(2):282–9. doi: 10.1016/j.gene.2014.04.077 (PMC4058751; doi:10.1016/j.gene.2014.04.077)
Supplement: Supplementary file 1 — Supplementary material. [file mmc1.doc]

**SUPPLEMENTARY MATERIAL**

**Figure S1. p53 in TF1 cells.**

Nuclear and cytoplasmic extracts were obtained from shSCR and shRPS19 TF1 cells. Immunoblot showed that p53 accumulates in the cytoplasm in these cells, especially when RPS19 is downregulated. Nu nuclear extract; Cy cytosolic extract.


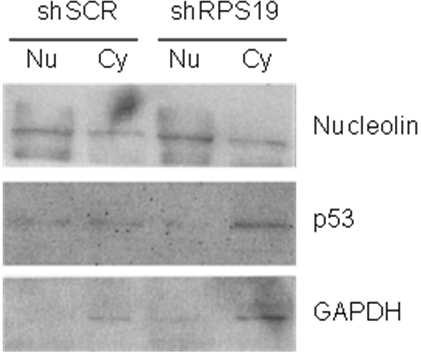


**Figure S2. Maturation markers on transduced TF1 cells.**

1. Transduction efficiency of the lentiviral vectors expressing shSCR or shRPS19 in a representative experiment.
2. Flow cytometry analysis showed no difference in the expression of selected surface markers between RPS19 downregulated cells and scramble control.

**
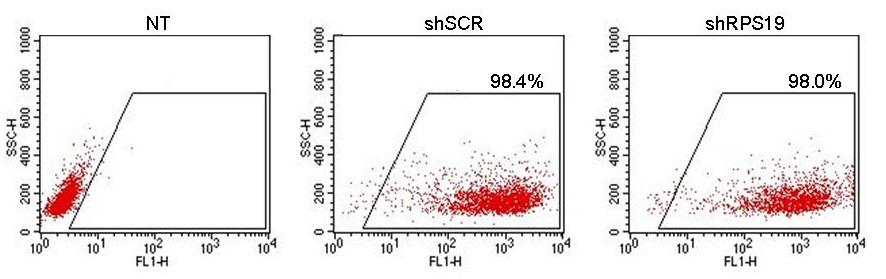
**

**A**

**
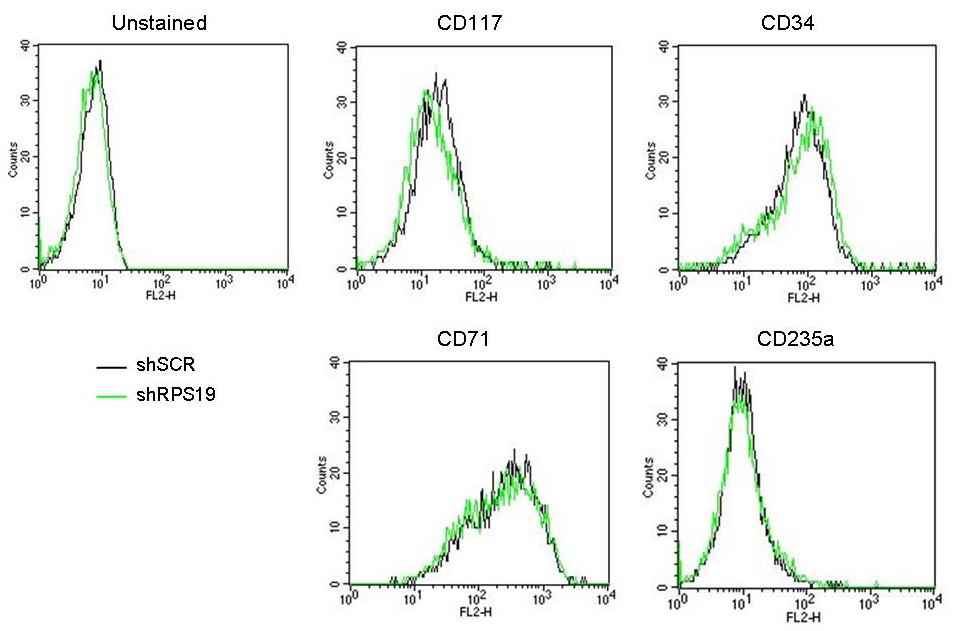
**

**B**

**Figure S3. RP silencing in TF1 cells.**

Western blot and densitometry showing the downregulation of RPL5 and RPL11 in TF1 cells compared to scramble controls (*p value < 0.05).

**
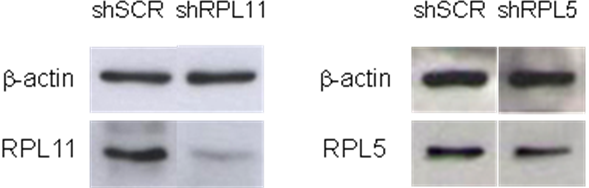
**

**
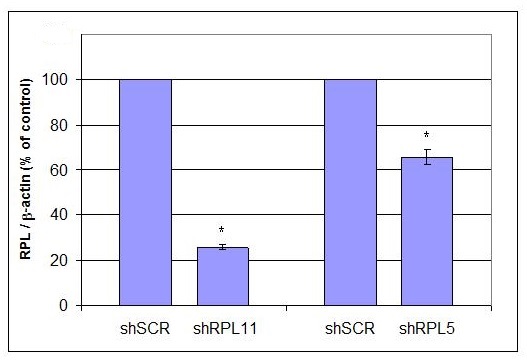
**

**Table S1.** Genes describing the first PC calculated on the dataset containing TF1 cell lines downregulated for RPS19, RPL5 or RPL11. FC: fold change values of the genes differentially expressed between silenced cells and scramble controls.

|  | **Probe ID** | **Gene Name** | **Gene Symbol** | **FC S19** | **FC L5** | **FC L11** |
| --- | --- | --- | --- | --- | --- | --- |
| 1 | 217809_at | basic leucine zipper and W2 domains 2 | BZW2 | 0.81 | 0.84 | 0.8 |
| 2 | 220059_at | signal transducing adaptor family member 1 | STAP1 | 1.4 | 1.74 | 1.76 |
| 3 | 211628_x_at | ferritin, heavy polypeptide pseudogene 1 | FTHL5 | 1.93 | 1.71 | 1.93 |
| 4 | 208693_s_at | glycyl-tRNA synthetase | Gars | 0.6 | 0.68 | 0.69 |
| 5 | 221524_s_at | Ras-related GTP binding D | Rragd | 0.66 | 0.6 | 0.58 |
| 6 | 200748_s_at | ferritin, heavy polypeptide 1; ferritin, heavy polypeptide-like 16; similar to ferritin, heavy polypeptide 1; ferritin, heavy polypeptide-like 3 pseudogene | FTHL3,FTHL16, FTHL20, FTH1 | 2.3 | 1.92 | 1.66 |
| 7 | 203186_s_at | S100 calcium binding protein A4 | S100a4 | 1.37 | 1.51 | 1.62 |
| 8 | 204485_s_at | target of myb1 (chicken)-like 1 | TOM1L1 | 0.56 | 0.46 | 0.38 |
| 9 | 201266_at | thioredoxin reductase 1; hypothetical LOC100130902 | LOC100130902, TXNRD1 | 0.71 | 0.58 | 0.55 |
| 10 | 201105_at | lectin, galactoside-binding, soluble, 1 | LGALS1 | 1.64 | 1.28 | 1.42 |
| 11 | 212877_at | kinesin light chain 1 | KLC1 | 0.6 | 0.6 | 0.45 |
| 12 | 218433_at | pantothenate kinase 3 | PANK3 | 0.59 | 0.58 | 0.62 |
| 13 | 206480_at | leukotriene C4 synthase | LTC4S | 1.8 | 2.85 | 2.99 |
| 14 | 203920_at | nuclear receptor subfamily 1, group H, member 3 | NR1H3 | 1.95 | 1.42 | 1.3 |
| 15 | 209122_at | adipose differentiation-related protein | PLIN2 | 1.59 | 1.79 | 2.33 |
| 16 | 203882_at | interferon regulatory factor 9 | IRF9 | 1.85 | 2.94 | 2.47 |
| 17 | 214909_s_at | dimethylarginine dimethylaminohydrolase 2 | ddah2 | 1.59 | 1.34 | 1.54 |
| 18 | 208813_at | glutamic-oxaloacetic transaminase 1, soluble (aspartate aminotransferase 1) | GOT1 | 0.63 | 0.5 | 0.43 |
| 19 | 200809_x_at | ribosomal protein L12 pseudogene 2; ribosomal protein L12 pseudogene 32; ribosomal protein L12 pseudogene 35; ribosomal protein L12 pseudogene 19; ribosomal protein L12 pseudogene 6; ribosomal protein L12; ribosomal protein L12 pseudogene 14 | RPL12P6,RPL12P32, RPL12P14, rpl12, RPL12P2, RPL12P35, RPL12P19 | 0.86 | 0.85 | 0.77 |
| 20 | 218237_s_at | solute carrier family 38, member 1 | SLC38A1 | 0.38 | 0.46 | 0.41 |
| 21 | 205277_at | PR domain containing 2, with ZNF domain | PRDM2 | 1.43 | 1.54 | 1.95 |
| 22 | 203661_s_at | tropomodulin 1 | TMOD1 | 1.25 | 1.38 | 1.32 |
| 23 | 212281_s_at | transmembrane protein 97 | TMEM97 | 0.6 | 0.73 | 0.59 |
| 24 | 202721_s_at | glutamine-fructose-6-phosphate transaminase 1 | GFPT1 | 0.77 | 0.61 | 0.54 |
| 25 | 211734_s_at | Fc fragment of IgE, high affinity I, receptor for; alpha polypeptide | FCER1A | 1.82 | 1.57 | 1.97 |
| 26 | 220892_s_at | chromosome 8 open reading frame 62; phosphoserine aminotransferase 1 | C8orf62 PSAT1 | 0.29 | 0.46 | 0.28 |
| 27 | 204859_s_at | apoptotic peptidase activating factor 1 | Apaf1 | 0.74 | 0.63 | 0.57 |
| 28 | 203127_s_at | serine palmitoyltransferase, long chain base subunit 2 | Sptlc2 | 0.68 | 0.64 | 0.49 |
| 29 | 211566_x_at | brain and reproductive organ-expressed (TNFRSF1A modulator) | BRE | 1.36 | 1.9 | 2.09 |
| 30 | 221666_s_at | PYD and CARD domain containing | pycard | 1.65 | 1.4 | 1.37 |
| 31 | 205077_s_at | phosphatidylinositol glycan anchor biosynthesis, class F | pigf | 1.73 | 1.48 | 1.79 |
| 32 | 202616_s_at | methyl CpG binding protein 2 (Rett syndrome) | mecp2 | 1.59 | 1.77 | 2.11 |
| 33 | 207039_at | cyclin-dependent kinase inhibitor 2A (melanoma, p16, inhibits CDK4) | CDKN2A | 1.29 | 1.36 | 1.51 |
| 34 | 203416_at | CD53 molecule | Cd53 | 1.43 | 1.55 | 1.71 |
| 35 | 220127_s_at | F-box and leucine-rich repeat protein 12 | fbxl12 | 1.27 | 1.25 | 1.32 |
| 36 | 219412_at | RAB38, member RAS oncogene family | RAB38 | 0.74 | 0.83 | 0.79 |
| 37 | 201761_at | methylenetetrahydrofolate dehydrogenase (NADP+ dependent) 2, methenyltetrahydrofolate cyclohydrolase | mthfd2 | 0.61 | 0.66 | 0.61 |
| 38 | 203355_s_at | pleckstrin and Sec7 domain containing 3 | PSD3 | 0.57 | 0.43 | 0.45 |
| 39 | 209263_x_at | tetraspanin 4 | tspan4 | 1.45 | 1.61 | 1.42 |
| 40 | 215127_s_at | RNA binding motif, single stranded interacting protein 1 | RBMS1 | 1.69 | 1.21 | 1.35 |
| 41 | 1729_at | TNFRSF1A-associated via death domain | TRADD | 1.28 | 1.31 | 1.34 |
| 42 | 219247_s_at | zinc finger, DHHC-type containing 14 | Zdhhc14 | 1.75 | 1.56 | 1.72 |
| 43 | 209644_x_at | cyclin-dependent kinase inhibitor 2A (melanoma, p16, inhibits CDK4) | CDKN2A | 1.14 | 1.17 | 1.18 |
| 44 | 205047_s_at | asparagine synthetase | asnS | 0.23 | 0.28 | 0.18 |
| 45 | 205632_s_at | phosphatidylinositol-4-phosphate 5-kinase, type I, beta | Pip5k1b | 1.45 | 1.33 | 1.49 |
| 46 | 221750_at | 3-hydroxy-3-methylglutaryl-Coenzyme A synthase 1 (soluble) | HMGCS1 | 0.5 | 0.45 | 0.34 |
| 47 | 31845_at | E74-like factor 4 (ets domain transcription factor) | Elf4 | 1.41 | 1.4 | 1.84 |
| 48 | 220560_at | chromosome 11 open reading frame 21 | C11orf21 | 1.41 | 2.07 | 1.87 |
| 49 | 218303_x_at | lysine-rich coiled-coil 1 | KRCC1 | 1.61 | 1.53 | 1.49 |
| 50 | 219092_s_at | inositol 1,3,4,5,6-pentakisphosphate 2-kinase | IPPK | 0.89 | 0.71 | 0.73 |
| 51 | 221269_s_at | SH3 domain binding glutamic acid-rich protein like 3 | SH3BGRL3 | 1.55 | 1.57 | 2.25 |
| 52 | 202022_at | aldolase C, fructose-bisphosphate | aldoc | 0.64 | 0.48 | 0.35 |
| 53 | 217127_at | cystathionase (cystathionine gamma-lyase) | CTH | 0.33 | 0.47 | 0.4 |
| 54 | 212645_x_at | brain and reproductive organ-expressed (TNFRSF1A modulator) | BRE | 1.39 | 1.69 | 2.07 |
| 55 | 210278_s_at | adaptor-related protein complex 4, sigma 1 subunit | ap4s1 | 1.55 | 1.95 | 1.95 |
| 56 | 209135_at | aspartate beta-hydroxylase | asph | 0.66 | 0.48 | 0.45 |
| 57 | 203057_s_at | PR domain containing 2, with ZNF domain | PRDM2 | 0.8 | 0.63 | 0.58 |
| 58 | 209301_at | carbonic anhydrase II | CA2 | 0.31 | 0.27 | 0.09 |
| 59 | 218859_s_at | similar to ABT1-associated protein; ESF1, nucleolar pre-rRNA processing protein, homolog (S. cerevisiae) | ESF1 | 0.62 | 0.51 | 0.47 |
| 60 | 205822_s_at | 3-hydroxy-3-methylglutaryl-Coenzyme A synthase 1 (soluble) | HMGCS1 | 0.41 | 0.28 | 0.15 |
| 61 | 204033_at | thyroid hormone receptor interactor 13 | Trip13 | 0.65 | 0.6 | 0.48 |
| 62 | 219366_at | apoptosis, caspase activation inhibitor | AVEN | 1.24 | 1.44 | 1.28 |
| 63 | 214271_x_at | ribosomal protein L12 pseudogene 2; ribosomal protein L12 pseudogene 32; ribosomal protein L12 pseudogene 35; ribosomal protein L12 pseudogene 19; ribosomal protein L12 pseudogene 6; ribosomal protein L12; ribosomal protein L12 pseudogene 14 | RPL12P6 RPL12P32 RPL12P14 rpl12 RPL12P2 RPL12P35 RPL12P19 | 0.86 | 0.86 | 0.83 |
| 64 | 212886_at | coiled-coil domain containing 69 | ccdc69 | 1.57 | 1.42 | 1.87 |
| 65 | 212333_at | family with sequence similarity 98, member A | Fam98a | 0.68 | 0.49 | 0.47 |
| 66 | 205453_at | homeobox B2 | HOXB2 | 1.24 | 1.23 | 1.19 |
| 67 | 212282_at | transmembrane protein 97 | TMEM97 | 0.65 | 0.72 | 0.54 |
| 68 | 200842_s_at | glutamyl-prolyl-tRNA synthetase | eprs | 0.68 | 0.48 | 0.4 |
| 69 | 207917_at | nudix (nucleoside diphosphate linked moiety X)-type motif 13 | Nudt13 | 0.63 | 0.71 | 0.59 |
| 70 | 218429_s_at | chromosome 19 open reading frame 66 | c19orf66 | 1.7 | 1.32 | 1.66 |
| 71 | 205252_at | zinc finger protein 174 | ZNF174 | 1.34 | 1.42 | 1.42 |
| 72 | 202722_s_at | glutamine-fructose-6-phosphate transaminase 1 | GFPT1 | 0.63 | 0.47 | 0.41 |
| 73 | 208030_s_at | adducin 1 (alpha) | Add1 | 1.28 | 1.42 | 1.59 |
| 74 | 205760_s_at | 8-oxoguanine DNA glycosylase | OGG1 | 1.56 | 1.9 | 2.16 |
| 75 | 212507_at | transmembrane protein 131 | TMEM131 | 1.33 | 1.35 | 1.38 |
| 76 | 202847_at | phosphoenolpyruvate carboxykinase 2 (mitochondrial) | pck2 | 0.65 | 0.63 | 0.5 |
| 77 | 218463_s_at | MUS81 endonuclease homolog (S. cerevisiae) | MUS81 | 1.2 | 1.24 | 1.31 |
| 78 | 208660_at | citrate synthase | CS | 0.83 | 0.76 | 0.77 |
| 79 | 204478_s_at | RAB interacting factor | Rabif | 1.31 | 1.51 | 1.75 |
| 80 | 208774_at | casein kinase 1, delta | CSNK1D | 1.33 | 1.63 | 1.87 |
| 81 | 215093_at | NAD(P) dependent steroid dehydrogenase-like | nsdhl | 0.78 | 0.66 | 0.59 |
| 82 | 218076_s_at | Rho GTPase activating protein 17 | arhgap17 | 0.82 | 0.7 | 0.68 |
| 83 | 201306_s_at | similar to Acidic leucine-rich nuclear phosphoprotein 32 family member B (PHAPI2 protein) (Silver-stainable protein SSP29) (Acidic protein rich in leucines); acidic (leucine-rich) nuclear phosphoprotein 32 family, member B | anp32b, LOC646791 | 0.72 | 0.89 | 0.84 |
| 84 | 215501_s_at | dual specificity phosphatase 10 | Dusp10 | 1.41 | 1.32 | 1.28 |
| 85 | 209331_s_at | MYC associated factor X | MAX | 1.47 | 1.21 | 1.29 |
| 86 | 202116_at | D4, zinc and double PHD fingers family 2 | dpf2 | 1.31 | 1.8 | 2 |
| 87 | 205550_s_at | brain and reproductive organ-expressed (TNFRSF1A modulator) | BRE | 1.4 | 1.84 | 2.3 |
| 88 | 202429_s_at | protein phosphatase 3 (formerly 2B), catalytic subunit, alpha isoform | ppp3ca | 1.32 | 1.35 | 1.45 |
| 89 | 202262_x_at | dimethylarginine dimethylaminohydrolase 2 | ddah2 | 1.65 | 1.37 | 1.52 |
| 90 | 208855_s_at | serine/threonine kinase 24 (STE20 homolog, yeast) | stk24 | 1.22 | 1.28 | 1.55 |
| 91 | 210277_at | adaptor-related protein complex 4, sigma 1 subunit | ap4s1 | 1.6 | 1.97 | 2.47 |
| 92 | 216999_at | erythropoietin receptor | EPOR | 1.76 | 1.41 | 1.91 |
| 93 | 203789_s_at | sema domain, immunoglobulin domain (Ig), short basic domain, secreted, (semaphorin) 3C | SEMA3C | 2.5 | 3.34 | 5.71 |
| 94 | 206085_s_at | cystathionase (cystathionine gamma-lyase) | CTH | 0.3 | 0.53 | 0.3 |
| 95 | 208315_x_at | TNF receptor-associated factor 3 | TRAF3 | 0.83 | 0.68 | 0.61 |
| 96 | 209486_at | UTP3, small subunit (SSU) processome component, homolog (S. cerevisiae) | UTP3 | 0.81 | 0.68 | 0.73 |
| 97 | 209549_s_at | deoxyguanosine kinase | Dguok | 1.27 | 1.24 | 1.24 |
| 98 | 204951_at | ras homolog gene family, member H | Rhoh | 1.38 | 2.85 | 3.43 |
| 99 | 202732_at | protein kinase (cAMP-dependent, catalytic) inhibitor gamma | PKIG | 1.27 | 1.86 | 1.96 |
| 100 | 206833_s_at | acylphosphatase 2, muscle type | Acyp2 | 1.3 | 1.59 | 1.67 |
| 101 | 218079_s_at | gametogenetin binding protein 2 | GGNBP2 | 1.27 | 1.26 | 1.52 |
| 102 | 213386_at | chromosome 9 open reading frame 125 | c9orf125 | 0.67 | 0.67 | 0.59 |
| 103 | 219933_at | glutaredoxin 2 | GLRX2 | 1.43 | 1.34 | 1.46 |
| 104 | 219215_s_at | solute carrier family 39 (zinc transporter), member 4 | SLC39A4 | 1.23 | 1.51 | 1.73 |
| 105 | 205662_at | B9 protein domain 1 | B9D1 | 0.78 | 0.58 | 0.53 |
| 106 | 208771_s_at | leukotriene A4 hydrolase | LTA4H | 0.47 | 0.78 | 0.67 |
| 107 | 213292_s_at | sorting nexin 13 | Snx13 | 0.7 | 0.62 | 0.47 |
| 108 | 215963_x_at | ribosomal protein L3 pseudogene 7 | RPL3P7 | 0.68 | 0.88 | 0.73 |
| 109 | 214639_s_at | homeobox A1 | hoxa1 | 1.84 | 1.83 | 1.85 |
| 110 | 212151_at | pre-B-cell leukemia homeobox 1 | PBX1 | 1.41 | 1.55 | 2.01 |
| 111 | 204039_at | CCAAT/enhancer binding protein (C/EBP), alpha | CEBPA | 0.64 | 0.64 | 0.5 |
| 112 | 203957_at | E2F transcription factor 6 | e2f6 | 0.83 | 0.73 | 0.65 |
| 113 | 220153_at | ectonucleoside triphosphate diphosphohydrolase 7 | ENTPD7 | 0.65 | 0.67 | 0.47 |
| 114 | 220615_s_at | fatty acyl CoA reductase 2 | FAR2 | 0.71 | 0.5 | 0.45 |
| 115 | 205812_s_at | transmembrane emp24 protein transport domain containing 9 | tmed9 | 0.89 | 0.75 | 0.72 |
| 116 | 212039_x_at | ribosomal protein L3; similar to 60S ribosomal protein L3 (L4) | RPL3 LOC653881 | 0.71 | 0.88 | 0.71 |
| 117 | 201196_s_at | adenosylmethionine decarboxylase 1 | AMD1 | 0.74 | 0.61 | 0.6 |
| 118 | 210044_s_at | lymphoblastic leukemia derived sequence 1 | Lyl1 | 1.26 | 1.57 | 1.56 |
| 119 | 221482_s_at | cAMP-regulated phosphoprotein 19 pseudogene; cAMP-regulated phosphoprotein, 19kDa | LOC646227 ARPP19 LOC643896 | 0.62 | 0.69 | 0.58 |
| 120 | 210357_s_at | spermine oxidase | SMOX | 1.43 | 1.38 | 1.77 |
| 121 | 201930_at | minichromosome maintenance complex component 6 | MCM6 | 0.6 | 0.77 | 0.59 |
| 122 | 202187_s_at | protein phosphatase 2, regulatory subunit B', alpha isoform | PPP2R5A | 1.43 | 1.42 | 1.54 |
| 123 | 201305_x_at | similar to Acidic leucine-rich nuclear phosphoprotein 32 family member B (PHAPI2 protein) (Silver-stainable protein SSP29) (Acidic protein rich in leucines); acidic (leucine-rich) nuclear phosphoprotein 32 family, member B | anp32b, LOC646791 | 0.71 | 0.83 | 0.84 |
| 124 | 204122_at | TYRO protein tyrosine kinase binding protein | TYROBP | 2.19 | 1.76 | 2.92 |
| 125 | 211423_s_at | sterol-C5-desaturase (ERG3 delta-5-desaturase homolog, S. cerevisiae)-like | SC5DL | 0.64 | 0.48 | 0.35 |
| 126 | 220486_x_at | hypothetical protein LOC100130886 |  | 3 | 1.57 | 2.57 |
| 127 | 204634_at | NIMA (never in mitosis gene a)-related kinase 4 | NEK4 | 0.7 | 0.54 | 0.47 |
| 128 | 207813_s_at | ferredoxin reductase | fdxr | 0.73 | 0.5 | 0.37 |
| 129 | 202617_s_at | methyl CpG binding protein 2 (Rett syndrome) | mecp2 | 1.67 | 1.75 | 2.66 |
| 130 | 203729_at | epithelial membrane protein 3 | emp3 | 1.38 | 1.46 | 1.94 |
| 131 | 211162_x_at | stearoyl-CoA desaturase (delta-9-desaturase) | scd | 0.62 | 0.41 | 0.37 |
| 132 | 200816_s_at | platelet-activating factor acetylhydrolase, isoform Ib, subunit 1 (45kDa) | Pafah1b1 | 1.36 | 1.37 | 1.48 |
| 133 | 219347_at | nudix (nucleoside diphosphate linked moiety X)-type motif 15 | NUDT15 | 0.7 | 0.69 | 0.63 |
| 134 | 211581_x_at | leukocyte specific transcript 1 | Lst1 | 1.29 | 1.41 | 1.64 |
| 135 | 209014_at | melanoma antigen family D, 1 | MAGED1 | 0.66 | 0.46 | 0.38 |
| 136 | 208691_at | transferrin receptor (p90, CD71) | TFRC | 1.17 | 1.21 | 1.26 |
| 137 | 215537_x_at | dimethylarginine dimethylaminohydrolase 2 | ddah2 | 1.61 | 1.37 | 1.56 |
| 138 | 208839_s_at | cullin-associated and neddylation-dissociated 1 | CAND1 | 0.83 | 0.65 | 0.59 |
| 139 | 200843_s_at | glutamyl-prolyl-tRNA synthetase | eprs | 0.72 | 0.7 | 0.54 |
| 140 | 209413_at | UDP-Gal:betaGlcNAc beta 1,4- galactosyltransferase, polypeptide 2 | b4galt2 | 0.71 | 0.86 | 0.8 |
| 141 | 201791_s_at | 7-dehydrocholesterol reductase | DHCR7 | 0.69 | 0.39 | 0.28 |
| 142 | 212740_at | phosphoinositide-3-kinase, regulatory subunit 4 | PIK3R4 | 0.66 | 0.72 | 0.83 |
| 143 | 209820_s_at | transducin (beta)-like 3 | tbl3 | 0.85 | 0.79 | 0.73 |
| 144 | 201818_at | lysophosphatidylcholine acyltransferase 1 | lpcat1 | 0.67 | 0.38 | 0.25 |
| 145 | 205078_at | phosphatidylinositol glycan anchor biosynthesis, class F | pigf | 2 | 1.38 | 1.42 |
| 146 | 201612_at | aldehyde dehydrogenase 9 family, member A1 | ALDH9A1 | 0.8 | 0.78 | 0.85 |
| 147 | 209382_at | polymerase (RNA) III (DNA directed) polypeptide C (62kD) | POLR3C | 1.4 | 1.19 | 1.3 |
| 148 | 202664_at | WAS/WASL interacting protein family, member 1 | WIPF1 | 1.42 | 1.4 | 1.57 |
| 149 | 211016_x_at | heat shock 70kDa protein 4 | HSPA4 | 0.87 | 0.59 | 0.57 |
| 150 | 203044_at | chondroitin sulfate synthase 1 | CHSY1 | 1.61 | 1.37 | 2.04 |
| 151 | 221529_s_at | plasmalemma vesicle associated protein | Plvap | 2.23 | 1.98 | 2.81 |
| 152 | 36742_at | tripartite motif-containing 15 | TRIM15 | 0.81 | 0.65 | 0.63 |
| 153 | 203846_at | tripartite motif-containing 32 | trim32 | 0.76 | 0.49 | 0.44 |
| 154 | 212825_at | PAX interacting (with transcription-activation domain) protein 1 | PAXIP1 | 0.68 | 0.48 | 0.47 |
| 155 | 205786_s_at | integrin, alpha M (complement component 3 receptor 3 subunit) | ITGAM | 1.82 | 1.92 | 1.91 |
| 156 | 209143_s_at | chloride channel, nucleotide-sensitive, 1A | CLNS1A | 0.71 | 0.82 | 0.68 |
| 157 | 212509_s_at | matrix-remodelling associated 7 | mxra7 | 0.77 | 0.74 | 0.63 |
| 158 | 209707_at | phosphatidylinositol glycan anchor biosynthesis, class K | PIGK | 2.12 | 1.74 | 2.88 |
| 159 | 218168_s_at | chaperone, ABC1 activity of bc1 complex homolog (S. pombe) | cabc1 | 0.72 | 0.87 | 0.82 |
| 160 | 219639_x_at | poly (ADP-ribose) polymerase family, member 6 | parp6 | 1.32 | 1.29 | 1.57 |
| 161 | 201876_at | paraoxonase 2 | PON2 | 0.72 | 0.49 | 0.53 |
| 162 | 201397_at | phosphoglycerate dehydrogenase | PHGDH | 0.29 | 0.6 | 0.53 |
| 163 | 336_at | thromboxane A2 receptor | tbxa2r | 2 | 1.26 | 2.05 |
| 164 | AFFX-r2-Bs-phe-3_at | Sporulation initiation phosphotransferase B | spo0B | 0.81 | 0.6 | 0.54 |
| 165 | 218321_x_at | serine/threonine/tyrosine interacting-like 1 | STYXL1 | 1.3 | 1.26 | 1.33 |
| 166 | 208117_s_at | LAS1-like (S. cerevisiae) | LAS1L | 0.84 | 0.73 | 0.64 |
| 167 | 202735_at | emopamil binding protein (sterol isomerase) | EBP | 0.79 | 0.71 | 0.59 |
| 168 | 209090_s_at | SH3-domain GRB2-like endophilin B1 | sh3glb1 | 1.26 | 1.73 | 1.56 |
| 169 | 201195_s_at | solute carrier family 7 (cationic amino acid transporter, y+ system), member 5 | SLC7A5 | 0.54 | 0.53 | 0.61 |
| 170 | 218870_at | Rho GTPase activating protein 15 | ARHGAP15 | 1.41 | 1.37 | 1.24 |
| 171 | 218106_s_at | mitochondrial ribosomal protein S10 | mrps10 | 1.72 | 1.7 | 2.65 |
| 172 | 218375_at | nudix (nucleoside diphosphate linked moiety X)-type motif 9 | nudt9 | 0.79 | 0.78 | 0.71 |
| 173 | 205171_at | protein tyrosine phosphatase, non-receptor type 4 (megakaryocyte) | PTPN4 | 0.77 | 0.66 | 0.58 |
| 174 | 210644_s_at | leukocyte-associated immunoglobulin-like receptor 1 | Lair1 | 1.82 | 1.56 | 2.05 |
| 175 | 208913_at | golgi associated, gamma adaptin ear containing, ARF binding protein 2 | Gga2 | 0.75 | 0.7 | 0.63 |
| 176 | 204295_at | surfeit 1 | SURF1 | 1.22 | 2.07 | 2.37 |
| 177 | 212290_at | solute carrier family 7 (cationic amino acid transporter, y+ system), member 1 | SLC7A1 | 0.69 | 0.72 | 0.6 |
| 178 | 205743_at | SH3 and cysteine rich domain | Stac | 1.39 | 2.54 | 3.55 |
| 179 | 204000_at | guanine nucleotide binding protein (G protein), beta 5 | GNB5 | 0.7 | 0.66 | 0.67 |
| 180 | 203177_x_at | transcription factor A, mitochondrial | TFAM | 0.75 | 0.67 | 0.57 |
| 181 | 38149_at | Rho GTPase activating protein 25 | Arhgap25 | 1.27 | 1.44 | 1.84 |
| 182 | 209383_at | DNA-damage-inducible transcript 3 | DDIT3 | 1.67 | 3.63 | 6.04 |
| 183 | 219013_at | UDP-N-acetyl-alpha-D-galactosamine:polypeptide N-acetylgalactosaminyltransferase 11 (GalNAc-T11) | galnt11 | 0.77 | 0.78 | 0.74 |
| 184 | 209146_at | sterol-C4-methyl oxidase-like | SC4MOL | 0.55 | 0.37 | 0.31 |
| 185 | 202886_s_at | protein phosphatase 2 (formerly 2A), regulatory subunit A, beta isoform | ppp2r1b | 0.78 | 0.68 | 0.6 |
| 186 | 205527_s_at | gem (nuclear organelle) associated protein 4 | GEMIN4 | 0.8 | 0.63 | 0.5 |
| 187 | 208647_at | farnesyl-diphosphate farnesyltransferase 1 | FDFT1 | 0.71 | 0.69 | 0.71 |
| 188 | 212242_at | tubulin, alpha 4a | TUBA4A | 0.65 | 0.54 | 0.32 |
| 189 | 208436_s_at | interferon regulatory factor 7 | IRF7 | 1.55 | 2.78 | 3.48 |
| 190 | 200868_s_at | ring finger protein 114 | RNF114 | 1.18 | 1.62 | 1.61 |
| 191 | 201576_s_at | galactosidase, beta 1 | Glb1 | 0.79 | 0.83 | 0.68 |
| 192 | 201477_s_at | ribonucleotide reductase M1 | Rrm1 | 0.63 | 0.83 | 0.74 |
| 193 | 215482_s_at | eukaryotic translation initiation factor 2B, subunit 4 delta, 67kDa | EIF2B4 | 1.4 | 1.48 | 1.21 |
| 194 | 215119_at | myosin XVI | Myo16 | 1.69 | 1.35 | 1.74 |
| 195 | 214749_s_at | similar to armadillo repeat containing, X-linked 6; armadillo repeat containing, X-linked 6 | LOC653354 Armcx6 | 1.44 | 1.77 | 2.05 |
| 196 | 216841_s_at | superoxide dismutase 2, mitochondrial | Sod2 | 0.77 | 0.8 | 0.83 |
| 197 | 208078_s_at | salt-inducible kinase 1 | Sik1 | 0.69 | 0.58 | 0.45 |
| 198 | 220973_s_at | SHANK-associated RH domain interactor | Sharpin | 1.37 | 1.58 | 1.84 |
| 199 | 213134_x_at | BTG family, member 3 | BTG3 | 0.67 | 0.36 | 0.24 |
| 200 | 209681_at | solute carrier family 19 (thiamine transporter), member 2 | SLC19A2 | 0.56 | 0.63 | 0.46 |
| 201 | 213625_at | zinc finger with KRAB and SCAN domains 4 | ZKSCAN4 | 1.3 | 1.58 | 2 |
| 202 | 219770_at | glycosyltransferase-like domain containing 1 | GTDC1 | 1.35 | 1.17 | 1.43 |
| 203 | 205169_at | retinoblastoma binding protein 5 | RBBP5 | 0.72 | 0.62 | 0.46 |
| 204 | 211582_x_at | leukocyte specific transcript 1 | Lst1 | 1.3 | 1.34 | 1.56 |
| 205 | 204882_at | Rho GTPase activating protein 25 | Arhgap25 | 1.4 | 1.35 | 1.91 |
